# The Role of Runx2 in Microtubule Acetylation in Bone Metastatic Breast Cancer Cells

**DOI:** 10.3390/cancers14143436

**Published:** 2022-07-15

**Authors:** Ahmad Othman, Marcus Winogradzki, Shreya Patel, Waddell Holmes, Alan Blank, Jitesh Pratap

**Affiliations:** 1 Department of Anatomy and Cell Biology, Rush University Medical Center, Suite 507, Armour Academic Building, Chicago, IL 60612, USA; ahmad.othman@northwestern.edu (A.O.); marcus_c_winogradzki@rush.edu (M.W.); shreya_patel@rush.edu (S.P.); waddell_b_holmes@rush.edu (W.H.); alan_blank@rush.edu (A.B.); 2 Division of Hematology/Oncology, Department of Medicine, Northwestern University Feinberg School of Medicine, Chicago, IL 60611, USA

**Keywords:** bone metastasis, breast cancer, microtubules, Runx2, tubulin acetylation, HDAC6

## Abstract

**Simple Summary:**

Breast cancer is the most commonly diagnosed cancer type, making up a quarter of all cases among women. While modern-day research has shown tremendous progress in the development of treatment options, complications related to bone metastasis remain an obstacle that demands further attention. Cancer cells that migrate into the bone microenvironment show significant alterations in their metabolism and gene expression profiles. In this study, we set out to better understand the changes induced by Runx2 in bone-derived breast cancer cells. We identified key changes in the stability of the microtubule cytoskeleton of bone-derived cells expressing Runx2. These changes have implications on a variety of processes related to metabolism, cellular stress response, and response to common chemotherapies. These studies help to shed light on the changes that take place as a tumor begins to metastasize and to better predict which therapies will benefit patients with bone metastasis.

**Abstract:**

Bone metastasis of breast cancer results in severe bone loss, fractures, and death. Crosstalk between breast cancer cells and bone resident cells promotes osteoclast activity and the release of growth factors from the bone matrix resulting in aggressive tumor growth and bone loss. We and others have shown that Runt-related transcription factor-2 (Runx2) promotes metastatic tumor growth-associated bone loss. Breast cancer cells also induce autophagy to survive metabolic stress at the metastatic site. Recently, we reported a Runx2-dependent increase in autophagy. In this study, to examine the underlying mechanisms of metastasis and tumor resistance to stress, we used a bone metastatic isogenic variant of breast cancer MDA-MB-231 cells isolated from a xenograft tumor mouse model of metastasis. Our results with immunofluorescence and biochemical approaches revealed that Runx2 promotes microtubule (MT) stability to facilitate autophagy. Stable MTs are critical for autophagosome trafficking and display increased acetylation at Lysine 40 of α-tubulin. Runx2 silencing decreases acetylated α-tubulin levels. The expression levels of HDAC6 and αTAT1, which serve to regulate the acetylation of α-tubulin, were not altered with Runx2 silencing. We found that HDAC6 interaction with α-tubulin is inhibited by Runt-related factor-2 (Runx2). We show that the expression of wild-type Runx2 can restore the acetylated polymer of MTs in Runx2 knockdown cells, while the C-terminal deletion mutant fails to rescue the polymer of MTs. Importantly, cellular stress, such as glucose starvation also increases the acetylation of α-tubulin. We found that the loss of Runx2 increases the sensitivity of breast cancer cells to MT-targeting agents. Overall, our results indicate a novel regulatory mechanism of microtubule acetylation and suggest that Runx2 and acetylated microtubules may serve as therapeutic targets for bone metastatic tumors.

## 1. Introduction

Bone metastasis is a frequent complication of breast cancer and a significant contributor to patient mortality. Despite recent advances in screening technologies and effective treatments, gaps remain in our knowledge of the mechanisms regulating the bone metastatic process [1,2,3,4,5].

Aberrant expression of Runx2 has been linked with the progression of breast cancer and development of bone metastasis through the transcriptional upregulation of genes including VEGF, MMP-2, 9, 13, and IL-6 among others, which serve to promote tumor cell migration and invasion. The C-terminal domain of Runx2 serves as a scaffold to regulate interactions with chromatin remodelers including HDAC6, p300, and others to facilitate DNA binding [6,7,8,9]. The scaffolding role of the C-terminus has been demonstrated to be critical for proper transcriptional regulation, with C-terminal deletion mutants displaying a dominant-negative phenotype. In addition to its role in the nucleus, Runx2 has also been observed in the cytoplasm colocalized with the microtubule cytoskeleton serving an unknown function [8,10].

Recently, we reported that Runx2 promotes autophagy without significant alterations in the core autophagy-related gene (ATG) expression in bone-derived breast cancer cells [3]. Autophagy represents a catabolic process whereby, in response to metabolic, chemical, or other stressors, cells engulf damaged organelles and proteins in double-membrane vesicles known as autophagosomes, which traffic to the lysosome for degradation into macromolecular subunits that can be used to fuel biosynthesis. In the context of an existing tumor, high levels of autophagy have been demonstrated to promote tumor growth and metastasis. Autophagosomes traffic along a subset of microtubules marked with acetylation at Lysine 40 of α-tubulin. Acetylated microtubules represent a stable fraction of microtubules that display decreased rates of catastrophe and enhanced bundling and compliance to facilitate vesicular traffic and resist the associated forces [3,11,12,13]. Acetylation occurs on the luminal surface of polymerized tubulin by α-tubulin N-acetyl transferase-1 (α-TAT-1), while deacetylation by HDAC6 favors oligomeric tubulin resulting from a catastrophe. Acetylation can dictate the recruitment of an assortment of microtubule-associated proteins (MAP), molecular motors, and others to regulate cellular activity and microtubule dynamics [3,14,15]. Similar to Runx2, high levels of autophagy and acetylated α-tubulin have been linked with resistance to anoikis, tumor progression, and metastasis, raising the question of the underlying mechanism of this phenotype [16]. In this study, we examined the role of Runx2 in tubulin acetylation and its impact on MTs’ stability, and the sensitivity of bone-derived breast cancer cells to microtubule-targeting agents. Our findings indicate that Runx2 enhances the α-tubulin stability of MTs and its knockdown sensitizes bone metastatic cells towards MT-targeting agents.

## 2. Materials and Methods

### 2.1. Cell Culture

Bone-derived metastatic MDA-MB-231 (BoM-MDA-231) control and Runx2 knockdown were isolated and cultured as previously described [2]. For nocodazole and vinblastine dose curve experiments, BoM-MDA-231 cells were seeded in 12-well plates and incubated overnight to reach confluence. Cells were washed with PBS and treated with control, nocodazole, or vinblastine at doses of 1 µM, 5 µM, and 10 µM for 4 h. Cells were then washed with PBS and collected in whole-cell lysis buffer for western blot analysis. For autophagy flux assessment, 10 µM doses of nocodazole and vinblastine were used in the presence and absence of hydroxychloroquine 50 µM (Sigma, St. Louis, MO, USA). Whole-cell lysates were collected for western blot analysis.

### 2.2. Immunofluorescence

Immunochemistry was performed on BoM-MDA-231 control and Runx2 knockdown as previously described [2,17]. To visualize acetylated microtubules, cells were seeded in 8-well glass chamber slides and incubated overnight. Cells were subjected to control, nocodazole, or vinblastine treatment followed by fixation and imaging. Primary antibodies for α-tubulin and acetylated α-tubulin (Lys40) were obtained from Cell Signaling Technologies (CST) and used at 1:1000 dilutions. Secondary anti-rabbit and anti-mouse antibodies were obtained from Invitrogen and used at 1:1000 dilutions. Anti-rabbit secondary antibody was conjugated with AF-594. Slides were mounted with prolonged antifade reagent with DAPI (Invitrogen, Waltham, MA, USA). Cells were imaged using the Zeiss (LSM70) Confocal microscope equipped with a 63X oil immersion lens. Images were quantified using ImageJ software. Quantification provides integrated density reflecting the intensity of the stain within the region. The data shown provide the mean, and error bars represent the SD.

### 2.3. Western Blotting

Whole-cell lysates were collected in the Laemelli sample buffer as previously described [18]; nuclear and cytoplasmic fractions were isolated as previously described [19]. Equal volumes of lysate were blotted and quantified as previously described [2,18,19,20]. Three pairs of lysates were used to determine the levels of detyrosinated α-tubulin (Abcam, Cambridge, UK), HDAC6, ATAT1 (Abcam), SIRT2 (CST), TGM2 (CST), and HSP90α (CST) expression levels. Whole-cell lysates were collected from BoM-MDA-231 cells following growth under standard culture conditions and blotted. For all western blots conducted using cytoplasmic and whole-cell lysate, β-actin (CST) was used as a loading control. Lamin B1 (Santa Cruz Biotechnology, Dallas, TX, USA) was utilized as a loading control for western blots involving nuclear protein fractions.

### 2.4. Co-Immunoprecipitation

Single-cell clones of virally-transduced BoM-MDA-231 cells expressing Runx2 shRNA were washed with ice-cold PBS, and then cells were lysed in 500 µL of immunoprecipitation buffer (20 mM Tris pH 7.5, 150 mM NaCl, 1 mM EDTA, 1 mM EGTA, 2.5 mM sodium pyrophosphate, 1 mM β-glycerophosphate, 1 mM sodium orthovanadate, 1% Triton X-100) supplemented with complete mini EDTA free protease inhibitor tablets (Roche) and 1 mM PMSF at the time of use. Lysates were then sonicated on ice for 20 s and centrifuged at 14,000× *g* for 10 min to pellet debris. Precleared lysates were centrifuged at 14,000× *g* for 10 min to pellet protein A/G beads (Santa Cruz Biotechnology). The resulting supernatant was equally divided into normal rabbit serum and was utilized as a negative control with a dilution of 1:200, and an HDAC6 antibody was utilized for IP at a dilution of 1:100; the resulting mixtures were then incubated overnight at 4 °C. Following incubation of 20 µL of protein, A/G beads were added to each reaction and incubated for an additional 3 h, the resulting mixture was then centrifuged and washed five times in 500 µL of IP buffer. Following the final wash, samples were centrifuged at 14,000× *g* for 10 min and the resulting pellet was suspended in 60 µL of 1× whole-cell lysis buffer, while inputs were mixed with the appropriate volume of 4× Laemelli buffer. The resulting samples were then blotted for α-tubulin and HDAC6.

### 2.5. Microtubule Polymer Mass Isolation

Microtubule polymer mass was isolated following a previously published protocol [21,22]. Cells were briefly washed with PBS and washed twice for 15 min at 37 °C in microtubule stabilization buffer (MTSB) (0.1 M PIPES pH 6.75, 1 mM EGTA pH 8, 1 mM MgSO_4_, 2 M Glycerol, 0.1% Triton X-100). To ensure reliable isolation, all buffers were warmed to 37 °C before use and supplemented with complete mini EDTA-free protease inhibitor tablets (Roche, Basel, Switzerland). Following washing, cells were collected in 1× sample buffer and prepared for western blotting analysis.

### 2.6. Viral Transduction

MDA-MB-231 cells were transduced with EV, wild-type Runx2 (WT), DNA Binding domain mutant Runx2 (DBD), and C-terminal deletion mutant Runx2 (ΔC) constructs as previously described [3,23]; 200 µL of virus solution was used for each vector EV, WT, DBD, and ΔC. Runx2 expression was verified by isolation of the nuclear protein followed by western blot. Due to low expression levels, transduced cells expressing Runx2 shRNA were serially diluted to generate single-cell clones. Runx2 expression was verified by western blot and optimal clones expressing either WT, DBD, or ΔC Runx2 were selected and used for all experiments [19,23].

### 2.7. qPCR Array Analysis

Total RNA from BoM-MDA-231 cells was isolated through column chromatography according to the manufacturer’s instructions (IBI Scientific, Dubuque, IA, USA) and quantified using a NanoDrop spectrophotometer (Thermo Fisher, Waltham, MA, USA). Total RNA samples were reverse transcribed according to the manufacturer’s instructions using superscript III reagents (Invitrogen). The resulting cDNA was used in subsequent PCR reactions. PCR arrays were obtained for human autophagy and cytoskeleton regulators (Qiagen PAHS-04Z, PAHS-088Z). PCR reactions were run on a Quant Studio 3 instrument (ABI) and analyzed according to array manufacturer instructions.

### 2.8. Statistical Analysis

Statistical analysis was conducted using GraphPad software. Unpaired *t*-tests were conducted using the mean and standard deviation for each assay. All graphs represent the mean + SD.

## 3. Results

**Figure 1:**
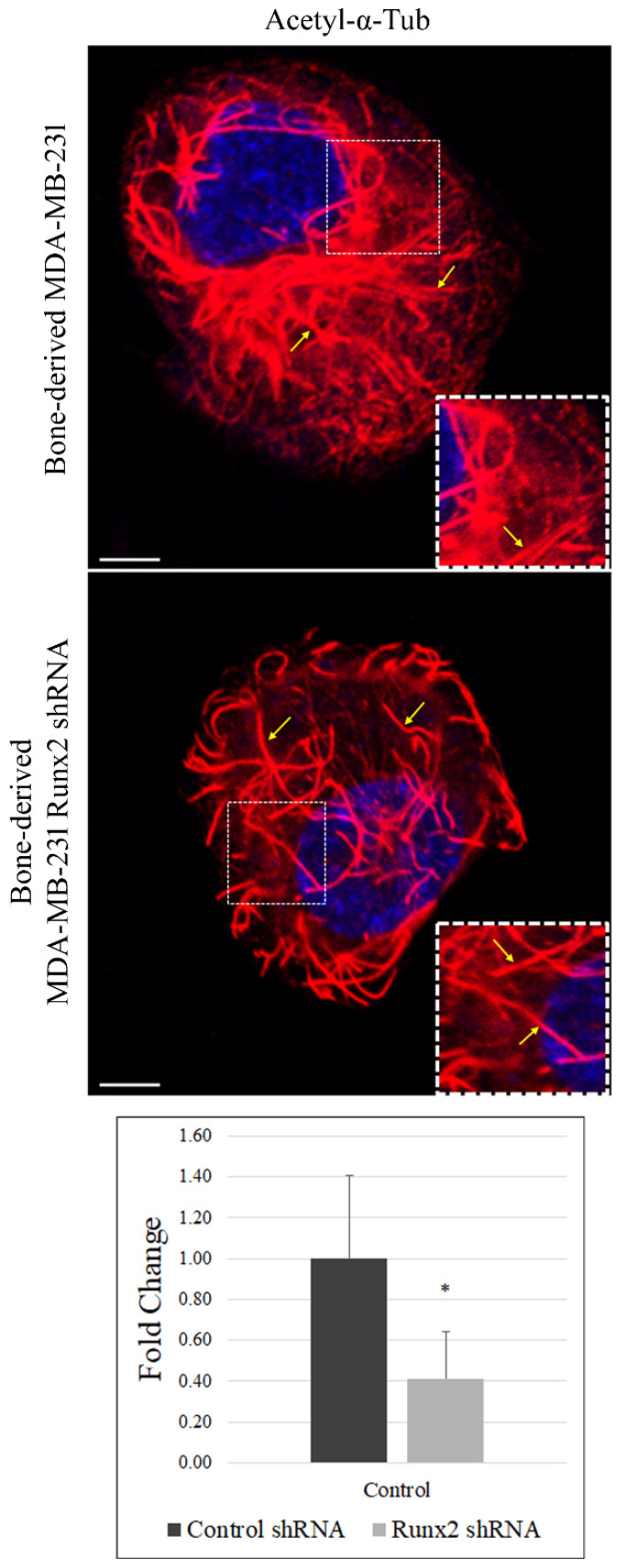
**Loss of Runx2 reduces α-tubulin acetylation in bone-derived MDA-MB-231 cells.** Immunofluorescence studies show reduced acetylation in the absence of Runx2 in BoM-MDA-231. The fixed cells were analyzed for acetyl-α-tubulin (Lys40) (Alexa fluor-594/Red). Acetyl-α-tubulin (Lys40) is stained red, while nuclei are stained blue with DAPI. The yellow arrows show tubular structures. Cells (*n* = 30) were quantified for tubulin signal intensity using ImageJ. Results were analyzed via an unpaired *t*-test (* *p* < 0.01). The scale bar represents 50 µm. The images shown are representative of 3 independent experiments.

The stable acetylated fraction of the microtubule cytoskeleton supports vesicular trafficking [14]. We reported that Runx2 facilitates autophagosome trafficking [3]. Based on these results, we examined the impact of Runx2 on stable microtubules. Confocal immunofluorescence microscopy for acetylated α-tubulin (Lys40) indicated that Runx2 silencing in BoM-MDA-231 cells resulted in reduced staining intensity as well as disrupted and discontinuous acetylated microtubules. A strong signal of perinuclear acetylated α-tubulin was observed in control cells that were disrupted with Runx2 silencing. ImageJ analysis revealed a significant reduction in acetylated α-tubulin signal intensity with Runx2 silencing (* *p* < 0.0001) (Figure 1).

**Figure 2:**
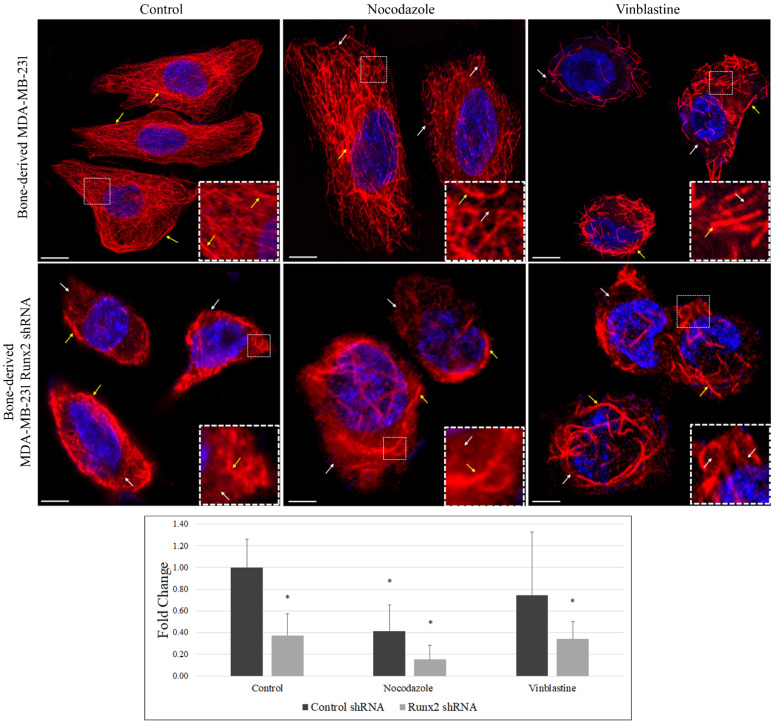
**Loss of Runx2 sensitizes α-tubulin acetylation in response to MT-targeting agents.** The bone-derived MDA-MB-231 control and Runx2 knockdown cells were treated with microtubule-targeting agents vinblastine (1 µM) and nocodazole (1 µM) for 5 min. The fixed cells were analyzed for acetyl-α-tubulin (Lys40) (Alexa fluor-594/Red) by immunofluorescence. Acetyl-α-tubulin (Lys40) is stained red, while nuclei are stained blue with DAPI. White arrows show loss of tubular structures and diffused staining. The yellow arrows show intact tubular structures. Cells (*n* = 30) were quantified for tubulin signal intensity using ImageJ. Results were analyzed via an unpaired *t*-test (* *p* < 0.0001). The scale bar represents 50 µm. The data shown are representative of 3 independent experiments.

Acetylated microtubules show enhanced stability and resistance to the depolymerizing agent nocodazole but not to vinblastine [14]. We examined whether Runx2 silencing impacts the microtubules’ stability. Immunostaining of BoM-MDA-231 cells treated with vinblastine (1 µM, 5 min) showed significantly reduced staining with Runx2 silencing relative to control cells (* *p* < 0.0001). Control cells treated with vinblastine displayed tangled and shortened microtubules, while Runx2 silencing resulted in a significant loss of signal with the presence of ring-shaped structures indicative of more advanced stages of microtubule catastrophe (Figure 2). These results suggest that a decrease in acetylated α-tubulin observed with Runx2 silencing is accompanied by reduced microtubule stability as observed with the vinblastine treatment.

**Figure 3:**
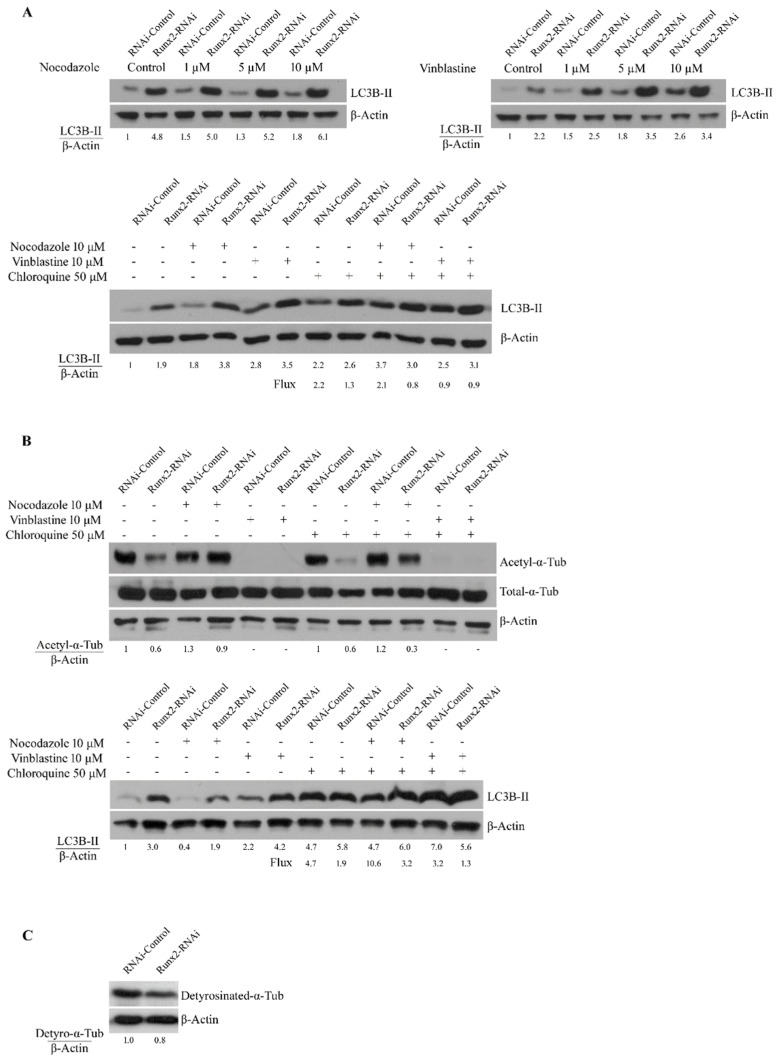
**Disruption of stable microtubules impacts autophagy.** (**A**) The western blots for LCB-II levels of whole-cell lysates from BoM-MDA-231 control and Runx2 knockdown cells treated with vinblastine and nocodazole. Cells were serum-starved overnight and were then treated with vinblastine and nocodazole for 3 h with indicated doses. The β-actin expression is shown as an internal loading control. (**B**) The western blots for acetyl-α-tubulin (Lys40) levels and LCB-II levels in BoM-MDA-231 control and Runx2 knockdown cells treated with nocodazole and vinblastine and subsequent washout. Cells were treated with nocodazole and vinblastine for 3 h and were then washed with PBS and replenished with standard culture media for an additional 3 h. Chloroquine treatment was present for the full duration of these experiments. Western blots shown are representative of 3 independent experiments. (**C**) Western blots showing the levels of detyrosinated α-tubulin in BoM-MDA-231 control and Runx2 knockdown cells.

Previous studies indicate that microtubule stability can alter the autophagy pathway [3,14]. To examine the effect of Runx2 on the dynamic and stable fractions of microtubules we performed vinblastine and nocodazole treatments and measured the levels of acetylated α-tubulin and autophagosome marker LC3B-II. Nocodazole targets the dynamic non-acetylated fraction of microtubules, while vinblastine targets all subsets of microtubules [14]. LC3B-II showed a robust accumulation with Runx2 silencing consistent with previous studies [3]. Interestingly, vinblastine treatment resulted in a dose-dependent increase in LC3B-II levels primarily in control cells, while nocodazole did not produce a change in levels (Figure 3a). To determine the autophagy flux, the lysosomal inhibitor chloroquine was utilized to measure rates of LC3B-II turnover. An analysis of autophagic flux showed minimal flux with Runx2 silencing, with vinblastine treatment resulting in a marked reduction in flux, while nocodazole treatment had minimal impact.

To determine whether the effects of nocodazole and vinblastine treatment were reversible, the treatments were followed by an equal washout period. Treatment with nocodazole followed by a washout period resulted in a robust increase in the acetylation of α-tubulin relative to the untreated controls, which were independent of the Runx2 status (Figure 3b upper panels). Vinblastine treatment and washout resulted in a complete loss of acetylated α-tubulin. Runx2 silencing reduced the acetylated α-tubulin levels relative to the control. Increased acetylation with the washout of nocodazole resulted in enhanced flux among control cells (4.7 to 10.6) and the restoration of flux in knockdowns (1.9 to 3.2), while the washout of vinblastine decreased the flux in both the control and knockdown cells (Figure 3b lower panel). Notably, a Runx2-dependent reduction in the acetylated α-tubulin levels was observed, specifically in BoM-MDA-231 cells, and no significant change was observed in parental MDA-MB-231 cells (Supplementary Figure S1).

In addition to acetylation, the detyrosination level of α-tubulin is another marker of microtubule stability [24]. We found a mild decrease in detyrosinated α-tubulin levels with Runx2 silencing as examined by western blot analysis (Figure 3c). Taken together these results suggest that Runx2 promotes microtubule stability and the acetylation levels of α-tubulin.

**Figure 4:**
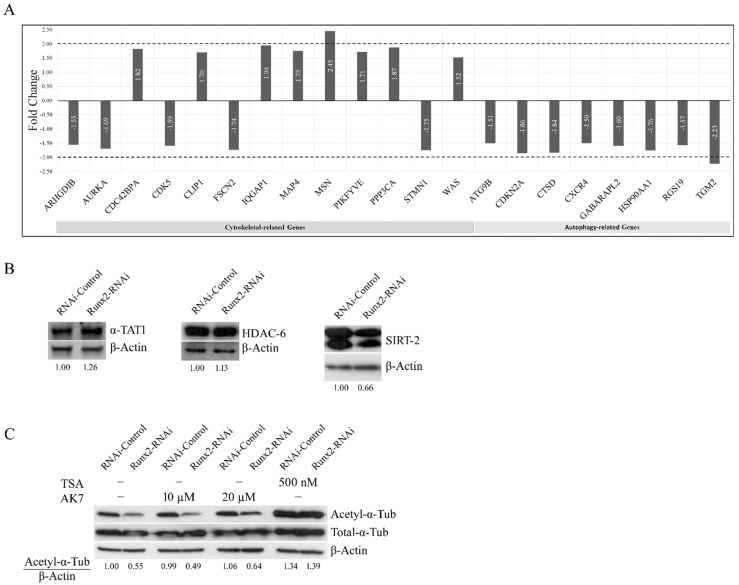
**Core autophagy-related genes and cytoskeleton regulatory genes are unchanged with Runx2 silencing in bone-derived MDA-MB-231 cells.** (**A**) qRT-PCR array analysis of autophagy and cytoskeletal-related genes in BoM-MDA-231 control and Runx2-RNAi cells. mRNA was reverse transcribed and profiled for these genes using PCR array #PAHS-088Z (84 cytoskeletal-related genes) and #PAHS-084Z (84 autophagy-related genes), results for both arrays were normalized to 5 housekeeping genes. (**B**) Western blot analysis shows the protein levels of the positive (alpha-tubulin acetyltransferase-1 (ATAT1)) and negative (HDAC6 and sirtuin2 (SIRT2)) regulators of α-tubulin acetylation in BoM-MDA-231 control and Runx2-RNAi cells. (**C**) α-tubulin acetylation levels in BoM-MDA-231 control and Runx2-RNAi cells treated with the pan histone deacetylase (HDAC) inhibitor trichostatin A (TSA) and the SIRT2 specific inhibitor AK7 for 24 h with indicated doses.

To determine the mechanism by which Runx2 promotes microtubule acetylation and autophagy, we performed a qRT-PCR array analysis for the expression of autophagy and cytoskeleton-related genes (a total of 168 genes, 84 from each pathway) in the control and Runx2-RNAi BoM-MDA-231 cells. Our analysis revealed that the expression of core autophagy regulators was largely unchanged with Runx2 silencing. Out of 84 genes examined, only transglutaminase 2 (TGM2) showed a greater than 2-fold downregulation with Runx2 silencing, which was further confirmed with western blotting (Figure 4a, Supplementary Figures S2 and S3a). However, TGM2 inhibition via LDN27219 revealed no change in α-tubulin acetylation. In the qRT-PCR array analysis of microtubule and actin cytoskeleton-related genes, we found a marginal change in the regulators of cytoskeletal dynamics with Runx2 silencing (Figure 4a). Interestingly, the actin-related gene Moesin (MSN) was upregulated 2.5-fold with Runx2 silencing. However, the western blot analysis of protein levels did not show significant changes (Supplementary Figure S3b).

**Figure 5:**
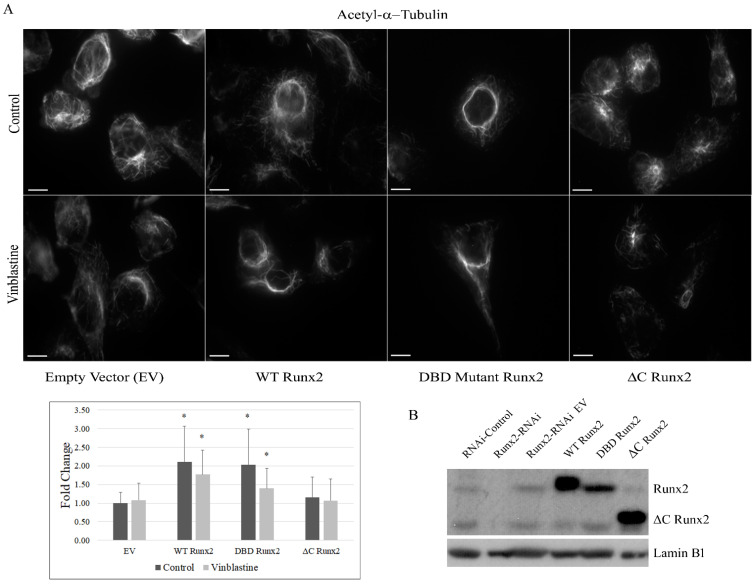
**Runx2 C-terminal is critical for acetyl-α-tubulin levels.** (**A**) Bone-derived MDA-MB-231 cells transduced with lentiviral constructs containing either empty vector (EV), wild-type Runx2, DNA binding domain mutant Runx2 (DBD Runx2), or C-terminal deletion Runx2 (ΔC-Runx2). Transduced cells were seeded overnight and were treated in the presence and absence of 1 mM vinblastine for 5 min. Cells were fixed and stained for acetyl-α-tubulin (Lys40) and imaged. Thirty cells per group were randomly selected and quantified for acetyl-α-tubulin signal intensity using ImageJ. Results were analyzed via unpaired t-test with comparison relative to corresponding EV control * *p* ≤ 0.05. Representative 100X images are shown. (**B**) Western blot analysis for Runx2 expression in nuclear lysates from the transduced cells. Lamin B1 levels were used as loading controls.

Next, we examined the protein levels of the positive regulator alpha-tubulin-acetyltransferase-1 (ATAT-1) and the negative regulators of α-tubulin acetylation HDAC6 and sirtuin2 (SIRT2) [25]. The whole-cell lysates from BoM-MDA-231 cells revealed no significant changes in HDAC6 and ATAT1 levels with Runx2 knockdown, while SIRT2 protein showed a mild reduction in response to Runx2 knockdown (Figure 4b). Treatment with the sirtuin2 specific inhibitor AK7 marginally increased the tubulin acetylation levels, while the pan HDAC inhibitor TSA produced a robust increase (Figure 4c), suggesting that HDAC6 is the primary deacetylase of a-tubulin in BoM-MDA-231 cells [3]. Altogether the qRT-PCR array analysis of the regulators of the cytoskeleton and autophagy pathways in BoM-MDA-231 showed that the core regulators of autophagy and cytoskeletal dynamics were unchanged with Runx2 silencing. Taken together with the protein levels of the regulators of α-tubulin acetylation, these results suggested a possibility of protein-protein interaction mechanisms of Runx2-mediated microtubule acetylation and stability. To examine this possibility, we generated a series of BoM-MDA-231 cell lines expressing empty vector construct (EV), wild-type Runx2 (WT), DNA binding domain mutant Runx2 (DBD), which lacks transcriptional activity, and a Runx2 C-terminal deletion mutant (ΔC), which can bind DNA but lacks interaction with other proteins. Immunofluorescence analysis showed significant increases in acetylation in response to WT and DBD Runx2 expression, while ΔC expression caused a significant decrease relative to EV (* *p* < 0.05). The expression of WT Runx2 significantly enhanced the resistance to vinblastine, while the expression of ΔC Runx2 caused a significant decrease (* *p* < 0.05) in vinblastine (Figure 5a). Morphologically, enhanced perinuclear bundling of acetylated α-tubulin was observed with WT and DBD expression, while ΔC caused diminished staining intensity and irregularities in the microtubular structure similar to observations in cells subjected to Runx2 silencing (Figure 5a). The expression levels of WT and mutant Runx2 proteins were confirmed with western blotting of nuclear lysates from these cells (Figure 5b).

**Figure 6:**
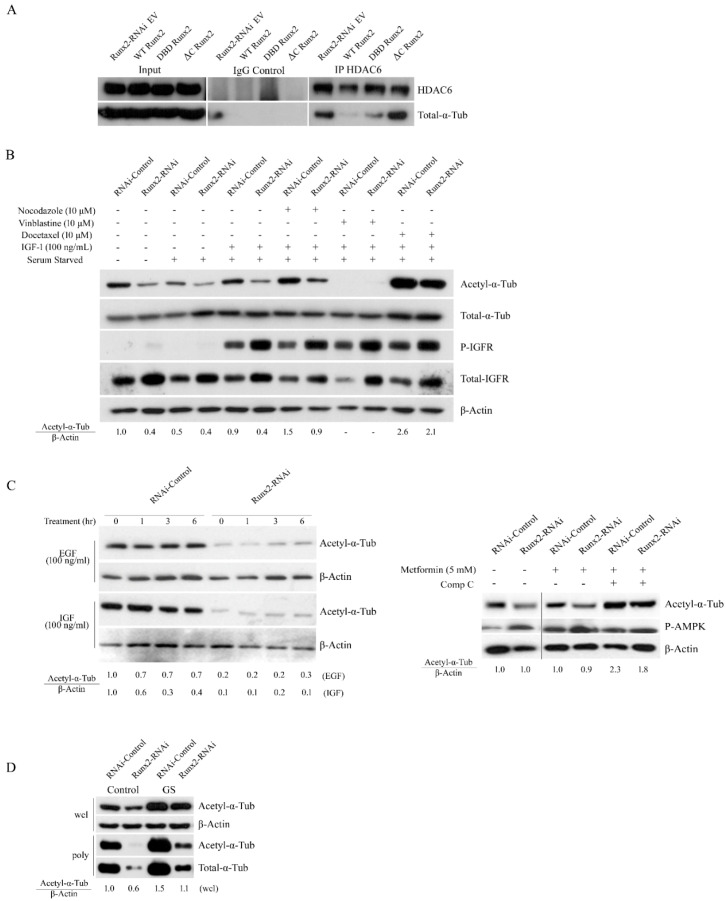
**Runx2 inhibits HDAC6 interaction with α-tubulin.** (**A**) Runx2 C-terminal suppresses the interaction of HDAC6 and α-tubulin. Input lysates from transduced BoM-MDA-231 Runx2-RNAi cells were collected followed by co-immunoprecipitation with either anti HDAC6 or control rabbit IgG. The resulting pellets were then blotted for HDAC6 and α-tubulin. (**B**) Western blot analysis for acetylated α-tubulin, total α-tubulin, phosphorylated IGF-1R, and total IGF-1R levels in whole-cell lysates from BoM-MDA-231 control and Runx2 knockdown cells treated with nocodazole, vinblastine, and docetaxel for 3 h followed by 3 h of washout. Cells were treated with IGF for 30 min before sample collection. The β-actin expression is shown as an internal loading control. (**C**) Western blot analysis of α-tubulin acetylation levels in BoM-MDA-231 and Runx2 knockdown cells treated with EGF (100 ng/mL) and IGF (100 ng/mL) for 0, 1, 3, and 6 h. The lower panel shows α-tubulin acetylation levels in cells that were treated with metformin (5 mM) or AMPK inhibitor compound C (5 µM) for 6 h. (**D**) α-tubulin acetylation and total tubulin levels in whole-cell lysates (wcl) and polymer mass (poly) of microtubules isolated from BoM-MDA-231 control and Runx2-RNAi cells. These cells were subjected to complete withdrawal of glucose (3 h) along with a parallel-group in which glucose was replenished (3 h) following withdrawal. Western blots shown are representative of 3 independent experiments.

Previous studies show that the C-terminal of Runx2 interacts with HDAC6 [3,9]. Our findings on the impact of HDAC inhibition and expression of the C-terminal deletion (ΔC Runx2) construct on tubulin acetylation and stability, and no change in HDAC6 expression with Runx2 knockdown led us to examine whether Runx2 sequestered HDAC6 from α-tubulin [3,9]. Immunoprecipitation of HDAC6 in transduced cells showed a substantial interaction between HDAC6 and α-tubulin in cells expressing EV and ΔC constructs but not WT or DBD (Figure 6a). These results suggest that Runx2 promotes acetylation by sequestering HDAC6 from α-tubulin.

Previous reports indicate crosstalk between microtubule dynamics and growth factor signaling or stress responses [26,27,28,29]; therefore, we examined the impact of microtubule-targeting agents (nocodazole, vinblastine, and docetaxel) on IGF-1R levels (Figure 6b). Consistent with our previous report [2], Runx2 knockdown cells show higher levels of pIGF-1R and total IGF-1R compared to controls. Interestingly, total IGF-1R levels were reduced with microtubule targeting agents, while no changes in pIGF-1R levels were observed. These results indicate that dynamic and stable fractions of microtubules are required for the maintenance of IGF-1R levels. Nocodazole treatment with no wash resulted in a mild increase in the acetylation of α-tubulin, while vinblastine treatment resulted in a complete loss of acetylated α-tubulin. Docetaxel treatment increased the acetylated α-tubulin levels independent of Runx2 levels. Furthermore, we found no significant changes in acetylated α-tubulin with 30 min treatment of IGF-1 in the control or Runx2 knockdown cells. Short-term treatments (1, 3, and 6 h) with IGF-1 or EGF showed no significant changes in the acetylation (Figure 6c). Next, we examined the impact of AMPK and glucose deprivation on acetylated α-tubulin levels. Activation of the AMPK pathway via metformin treatment also did not change the acetylation levels. Interestingly, inhibition of the AMPK pathway via compound C increased the acetylation to similar levels in the control and Runx2 knockdown cells (Figure 6c lower panel). To evaluate the impact of glucose on acetyl α-tubulin, BoM-MDA-231 cells were subjected to the complete withdrawal of glucose. As previously shown, Runx2 knockdown resulted in a 36% reduction in levels of acetyl α-tubulin relative to control cells; interestingly the complete removal of glucose for 3 h served as a potent inducer of α-tubulin acetylation in both the control and Runx2 knockdown cells (Figure 6d). These results indicate that glucose starvation can increase microtubule stability.

**Figure 7:**
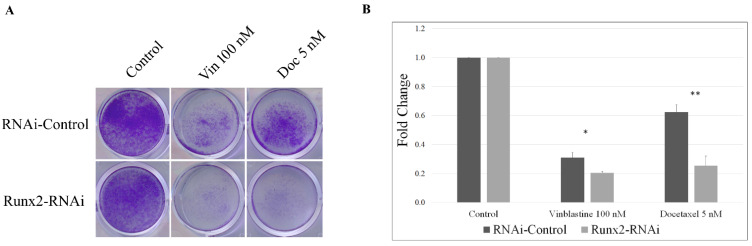
**Runx2 silencing increases sensitivity to microtubule-targeting agents.** (**A**) Bone-derived MDA-MB-231 control and Runx2 knockdown cells were seeded at 10% confluency for day 0, treated with either vinblastine 100 nM or docetaxel 5 nM for 1 h on both day 1 and day 3, and crystal violet-stained day 6. Shown above is a representative image (*n* = 3). Results were analyzed via an unpaired *t*-test (* *p* < 0.05, ** *p* < 0.005). (**B**) shows the fold change in the staining intensity (*n* = 3).

Finally, to understand the phenotypic changes due to Runx2-regulated microtubule stability, we examined whether Runx2 levels can impact the sensitivity of BoM-MDA-231 cells to microtubule-targeting agents. We treated the controls and Runx2 knockdown cells with either vinblastine (100 nM) or docetaxel (5 nM) for 1 h on both day 1 and day 3 and stained them with crystal violet on day 6. Our results show that knockdown of Runx2 significantly increases the sensitivity of these cells to vinblastine and docetaxel treatments (Figure 7).

## 4. Discussion

**Figure 8:**
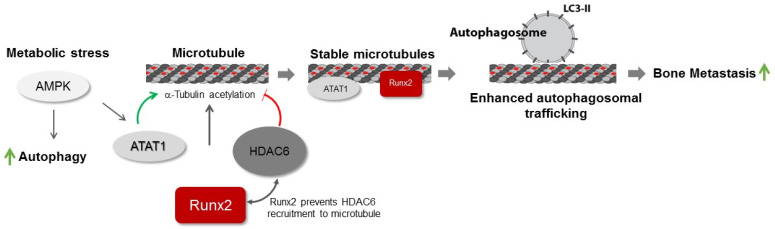
The main factors and pathways contributing to microtubule stability and acetylation in bone metastasis are shown. The AMPK pathway positively regulates autophagy and α-tubulin N-acetyl transferase-1 (ATAT-1). Acetylation of α-tubulin is increased by ATAT-1 and can be reversed by HDAC6. Our results show that Runx2 promotes acetylation of α-tubulin and microtubule stability. We found that Runx2 inhibits HDAC6 interaction with α-tubulin resulting in increased acetylation and stability of microtubules and enhanced autophagy flux. Previous reports show that upregulation of the autophagy pathway supports tumor growth and bone metastasis.

Runx2 is a key regulator of skeletal development. In the pathological context, Runx2 can promote metastatic properties and associated tumor-mediated osteolysis [2,30]. Previous reports show that Runx2 co-localizes with the microtubule cytoskeleton and that the Runx2 C-terminus interacts with HDAC6 [9,10]. Our findings suggest that Runx2 promotes α-tubulin acetylation and MTs’ stability in bone metastatic MDA-MB-231 cells. Previous studies suggest that interactions between Runx2 and the microtubule cytoskeleton can serve as a conduit to the nucleus to promote transcriptional activity. In vitro studies show that the interaction between Runx2 and HDAC6 can change the chromatin landscape and induce subsequent changes in gene expression [9,10]. However, the impact of these interactions on the microtubule cytoskeleton is not clear. Our results suggest that interactions among Runx2, microtubules, and HDAC6 can regulate the acetylation and stability of the microtubule cytoskeleton (Figure 8). We found increased microtubule acetylation with the expression of WT and DBD Runx2 constructs but not ΔC mutant, suggesting that Runx2 can serve a scaffolding role to promote microtubule stability possibly through sequestering the microtubule-associated deacetylase HDAC6. Previous studies have shown the interaction of Runx2 with multiple proteins including SMADs and chromatin remodelers such as p300 and HDACs to promote DNA binding [31]. Our findings highlight the possibility of Runx2-dependent recruitment of key microtubule associate proteins (MAPs) to the microtubules.

Acetylated α-tubulin levels and the autophagy pathway have been linked with resistance to anoikis, tumor progression, and metastasis [16,32,33]. Our results of Runx2-mediated tubulin acetylation revealed a novel mechanism by which Runx2 can promote the bone metastatic phenotype in addition to regulating cancer-related genes. Together these studies support the possibility of Runx2 serving a scaffolding role in microtubule dynamics in bone metastatic cancer cells.

We found that glucose deprivation induces microtubule acetylation in both the control and Runx2 knockdown cells. The underlying mechanism may involve glucose deprivation-dependent upregulation of AMPK signaling. Furthermore, our prior studies have demonstrated enhanced AMPK signaling in response to Runx2 silencing [2]. In response to stress, the AMPK activity can increase the expression and phosphorylation of ATAT1 and decrease HDAC6 levels, which correlate with enhanced α-tubulin acetylation and autophagic flux [15,34]. While ATAT1 levels were unchanged with Runx2 silencing, our study did not examine the effects of long-term starvation, which would allow for sufficient time to observe changes in protein expression. These results suggest crosstalk between AMPK signaling in response to Runx2 silencing and the cytoskeletal dynamics. We found that during glucose starvation conditions, α-tubulin acetylation was increased in controls and Runx2 shRNA cells at similar levels suggesting multiple mechanisms for tubulin acetylation.

ERK has been demonstrated to interact with a variety of MAP proteins and regulate their activity through phosphorylation events, which in turn could be the cause of the instability observed. MCF-10A breast epithelial cells and 10T1/2 fibroblasts expressing the mutant H-ras construct displayed significant decreases in levels of acetylated α-tubulin, which could be rescued through treatment with the MEK inhibitor PD98059 or transfection of a dominant-negative MEK construct. Furthermore, the expression of mutant ras resulted in enhanced co-localization of ERK with the microtubule cytoskeleton [35]. Interestingly, Runx2 has been shown to positively regulate the expression of β-Arrestin resulting in reduced ERK activity with Runx2 silencing [2]. Combined these findings suggest that while alterations in AMPK and MAPK signaling may be able to induce changes in microtubule dynamics, short-term treatments are not the cause of the instability observed with Runx2 silencing. Our study provides evidence of a novel role of Runx2 in the regulation of microtubule stability in cancer cells. The impact of AMPK and other signaling pathways on microtubule dynamics along with cytoskeletal responses to chronic vs. acute stress remains unknown and needs further investigation. The Runx2-dependent microtubule acetylation and its impact on autophagy may help elucidate new mechanisms of breast cancer bone metastatic cell function.

## Data Availability

The data presented in this study are available in this article (and supplementary material).

## Supplementary Materials

The following supporting information can be downloaded at: https://www.mdpi.com/article/10.3390/cancers14143436/s1, Figure S1: Acetyl-α-tubulin protein levels in parental and BoM-MDA-231 cells. Whole-cell lysates were collected from these cells grown under standard culture conditions. The β-actin expression is shown as an internal loading control. Figure S2: (**A**): Complete list of qRT-PCR array analysis of human cytoskeletal-related genes in BoM-MDA-231 control and Runx2-RNAi cells. mRNA was reverse transcribed and profiled for these genes using PCR array #PAHS-088Z (84 cytoskeletal-related genes); results were normalized to 5 housekeeping genes. (**B**) Complete list of qRT-PCR array analysis of human autophagy-related genes in BoM-MDA-231 control and Runx2-RNAi cells. mRNA was reverse transcribed and profiled for these genes using PCR array #PAHS-084Z (84 autophagy-related genes); results were normalized to 5 housekeeping genes. Figure S3: (**A**) TGM2 inhibition does not alter levels of acetyl-α-tubulin. Western blot analysis was performed on whole-cell lysates obtained from BoM-MDA-231. Cells were washed with PBS and treated with LDN27219 (10 µM) for 2 h and collected in lysis buffer and blotted for acetyl-α-tubulin and α-tubulin, and quantified with normalization to β-actin levels. Significance was calculated using an unpaired *t*-test. (**B**) Moesin protein levels in BoM-MDA-231. Whole-cell lysates were collected from four pairs of BoM-MDA-231 cells grown under standard culture conditions. Lysates were blotted for Moesin and quantified with normalization to β-actin levels. Significance was calculated using an unpaired *t*-test.
