# Supplementary material for: The Role of Runx2 in Microtubule Acetylation in Bone Metastatic Breast Cancer Cells
Source: Cancers (Basel). 2022 Jul 15;14(14):3436. doi: 10.3390/cancers14143436 (PMC9323014; doi:10.3390/cancers14143436)

Supplemental Figure S1

A

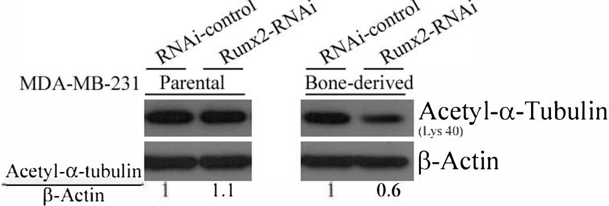

Supplemental Figure S2

A: HMN Cytoskeletal Array
Catalog #: PAHS-088Z

Unigene	Refseq	Symbol	Description	Gname	RT2 Catalog
Hs.643727	NM_005722	ACTR2	ARP2 actin-related protein 2 homolog (yeast)	ARP2	PPH02650A
Hs.433512	NM_005721	ACTR3	ARP3 actin-related protein 3 homolog (yeast)	ARP3	PPH02648A
Hs.503165	NM_015242	ARAP1	ArfGAP with RhoGAP domain, ankyrin repeat and PH domain 1	CENTD2	PPH14047A
Hs.75139	NM_012402	ARFIP2	ADP-ribosylation factor interacting protein 2	POR1	PPH09936A
Hs.435291	NM_013423	ARHGAP6	Rho GTPase activating protein 6	RHOGAP6/RHOGAPX-1	PPH15096F
Hs.504877	NM_001175	ARHGDIB	Rho GDP dissociation inhibitor (GDI) beta	D4/GDIA2/GDID4/LYGDI/Ly-GDI/RAP1GN1/RhoGDI2	PPH00010F
Hs.516954	NM_198236	ARHGEF11	Rho guanine nucleotide exchange factor (GEF) 11	GTRAP48/PDZ-RHOGEF	PPH18149A
Hs.489284	NM_005720	ARPC1B	Actin related protein 2/3 complex, subunit 1B, 41kDa	ARC41/p40-ARC/p41-ARC	PPH20196A
Hs.529303	NM_005731	ARPC2	Actin related protein 2/3 complex, subunit 2, 34kDa	ARC34/PNAS-139/PRO2446/p34-Arc	PPH10281B
Hs.524741	NM_001278556	ARPC3	Actin related protein 2/3 complex, subunit 3, 21kDa	ARC21/p21-Arc	PPH20880A
Hs.323342	NM_005718	ARPC4	Actin related protein 2/3 complex, subunit 4, 20kDa	ARC20/P20-ARC	PPH16919A
Hs.518609	NM_005717	ARPC5	Actin related protein 2/3 complex, subunit 5, 16kDa	ARC16/dJ127C7.3/p16-Arc	PPH11753A
Hs.250822	NM_003600	AURKA	Aurora kinase A	AIK/ARK1/AURA/AURORA2/BTAK/PPP1R47/STK15/STK6/STK7	PPH15095A
Hs.442658	NM_004217	AURKB	Aurora kinase B	AIK2/AIM-1/AIM1/ARK2/AurB/IPL1/PPP1R48/STK12/STK5/aurkb-sv1/aurkb-sv2	PPH21059F
Hs.98338	NM_003160	AURKC	Aurora kinase C	AIE2/AIK3/ARK3/AurC/HEL-S-90/SPGF5/STK13/aurora-C	PPH10708A
Hs.128316	NM_006340	BAIAP2	BAI1-associated protein 2	BAP2/FLAF3/IRSP53	PPH11890A
Hs.490203	NM_004342	CALD1	Caldesmon 1	CDM/H-CAD/HCAD/L-CAD/LCAD/NAG22	PPH21139A
Hs.282410	NM_006888	CALM1	Calmodulin 1 (phosphorylase kinase, delta)	CALML2/CAMI/CPVT4/DD132/LQT14/PHKD/caM	PPH01491C
Hs.495984	NM_003688	CASK	Calcium/calmodulin-dependent serine protein kinase (MAGUK family)	CAGH39/CAMGUK/CMG/FGS4/LIN2/MICPCH/MRXSNA/TNRC8	PPH16129A
Hs.417050	NM_003914	CCNA1	Cyclin A1	CT146	PPH00971A
Hs.194698	NM_004701	CCNB2	Cyclin B2	HsT17299	PPH00937F
Hs.467637	NM_001791	CDC42	Cell division cycle 42 (GTP binding protein, 25kDa)	CDC42Hs/G25K	PPH00729E
Hs.35433	NM_003607	CDC42BPA	CDC42 binding protein kinase alpha (DMPK-like)	MRCK/MRCKA/PK428	PPH08185B
Hs.343380	NM_006779	CDC42EP2	CDC42 effector protein (Rho GTPase binding) 2	BORG1/CEP2	PPH07846A
Hs.369574	NM_006449	CDC42EP3	CDC42 effector protein (Rho GTPase binding) 3	BORG2/CEP3/UB1	PPH17631B
Hs.647078	NM_004935	CDK5	Cyclin-dependent kinase 5	LIS7/PSSALRE	PPH02061E
Hs.500015	NM_003885	CDK5R1	Cyclin-dependent kinase 5, regulatory subunit 1 (p35)	CDK5P35/CDK5R/NCK5A/p23/p25/p35/p35nck5a	PPH00951A
Hs.170622	NM_005507	CFL1	Cofilin 1 (non-muscle)	CFL/HEL-S-15/cofilin	PPH13461F
Hs.119594	NM_007174	CIT	Citron (rho-interacting, serine/threonine kinase 21)	CRIK/STK21	PPH23914A
Hs.469840	NM_015282	CLASP1	Cytoplasmic linker associated protein 1	MAST1	PPH20650A
Hs.108614	NM_015097	CLASP2	Cytoplasmic linker associated protein 2	-	PPH58072B
Hs.524809	NM_002956	CLIP1	CAP-GLY domain containing linker protein 1	CLIP/CLIP-170/CLIP170/CYLN1/RSN	PPH01160A
Hs.647018	NM_003388	CLIP2	CAP-GLY domain containing linker protein 2	CLIP/CLIP-115/CYLN2/WBSCR3/WBSCR4/WSCR3/WSCR4	PPH10952B
Hs.461896	NM_016823	CRK	V-crk sarcoma virus CT10 oncogene homolog (avian)	CRKII/p38	PPH00731A
Hs.596164	NM_005231	CTTN	Cortactin	EMS1	PPH05698B
Hs.26704	NM_014608	CYFIP1	Cytoplasmic FMR1 interacting protein 1	P140SRA-1/SHYC/SRA-1/SRA1	PPH08693A
Hs.519702	NM_014376	CYFIP2	Cytoplasmic FMR1 interacting protein 2	PIR121	PPH14474F
Hs.529451	NM_005219	DIAPH1	Diaphanous homolog 1 (Drosophila)	DFNA1/DIA1/DRF1/LFHL1/hDIA1	PPH20113B
Hs.304192	NM_006870	DSTN	Destrin (actin depolymerizing factor)	ACTDP/ADF/HEL32/bA462D18.2	PPH19416A
Hs.487027	NM_003379	EZR	Ezrin	CVIL/CVL/HEL-S-105/VIL2	PPH00792B
Hs.134060	NM_017737	FNBP1L	Formin binding protein 1-like	C1orf39/TOCA1	PPH14842A
Hs.118555	NM_012418	FSCN2	Fascin homolog 2, actin-bundling protein, retinal (Strongylocentrotus purpuratus)	RFSN/RP30	PPH05554A
Hs.522373	NM_000177	GSN	Gelsolin	ADF/AGEL	PPH02146C
Hs.430551	NM_003870	IQGAP1	IQ motif containing GTPase activating protein 1	HUMORFA01/SAR1/p195	PPH00734A
Hs.291030	NM_006633	IQGAP2	IQ motif containing GTPase activating protein 2	-	PPH18229B
Hs.647035	NM_002314	LIMK1	LIM domain kinase 1	LIMK/LIMK-1	PPH01081A
Hs.474596	NM_005569	LIMK2	LIM domain kinase 2	-	PPH15654A
Hs.513983	NM_004140	LLGL1	Lethal giant larvae homolog 1 (Drosophila)	DLG4/HUGL/HUGL-1/HUGL1/LLGL	PPH21268A
Hs.472475	NM_012090	MACF1	Microtubule-actin crosslinking factor 1	ABP620/ACF7/MACF/OFC4	PPH18192A
Hs.502872	NM_002419	MAP3K11	Mitogen-activated protein kinase kinase kinase 11	MEKK11/MLK-3/MLK3/PTK1/SPRK	PPH00751B
Hs.517949	NM_002375	MAP4	Microtubule-associated protein 4	-	PPH00381A
Hs.178695	NM_002754	MAPK13	Mitogen-activated protein kinase 13	MAPK 13/MAPK-13/PRKM13/SAPK4/p38delta	PPH00188C
Hs.472437	NM_012325	MAPRE1	Microtubule-associated protein, RP/EB family, member 1	EB1	PPH19426A
Hs.532824	NM_014268	MAPRE2	Microtubule-associated protein, RP/EB family, member 2	EB1/EB2/RP1	PPH21359A
Hs.101174	NM_005910	MAPT	Microtubule-associated protein tau	DDPAC/FTDP-17/MAPTL/MSTD/MTBT1/MTBT2/PPND/PPP1R103/TAU	PPH05972F
Hs.567261	NM_004954	MARK2	MAP/microtubule affinity-regulating kinase 2	EMK-1/EMK1/PAR-1/Par-1b/Par1b	PPH13048A
Hs.27695	NM_000381	MID1	Midline 1 (Opitz/BBB syndrome)	BBBG1/FXY/GBBB1/MIDIN/OGS1/OS/OSX/RNF59/TRIM18/XPRF/ZNFXY	PPH08761B
Hs.713679	NM_002444	MSN	Moesin	HEL70	PPH13452B
Hs.477375	NM_053025	MYLK	Myosin light chain kinase	AAT7/KRP/MLCK/MLCK1/MLCK108/MLCK210/MSTP083/MYLK1/smMLCK	PPH18785B
Hs.86092	NM_033118	MYLK2	Myosin light chain kinase 2	KMLC/MLCK/MLCK2/skMLCK	PPH10337A
Hs.477693	NM_006153	NCK1	NCK adaptor protein 1	NCK/NCKalpha/nck-1	PPH00736A
Hs.529244	NM_003581	NCK2	NCK adaptor protein 2	GRB4/NCKbeta	PPH01626B
Hs.435714	NM_002576	PAK1	P21 protein (Cdc42/Rac)-activated kinase 1	PAKalpha	PPH01505F
Hs.20447	NM_005884	PAK4	P21 protein (Cdc42/Rac)-activated kinase 4	-	PPH08265A
Hs.91747	NM_002628	PFN2	Profilin 2	D3S1319E/PFL	PPH10486B
Hs.477114	NM_145753	PHLDB2	Pleckstrin homology-like domain, family B, member 2	LL5b/LL5beta	PPH07509A
Hs.744997	NM_015040	PIKFYVE	Phosphoinositide kinase, FYVE finger containing	CFD/FAB1/HEL37/PIP5K/PIP5K3/ZFYVE29	PPH13077B
Hs.49582	NM_002480	PPP1R12A	Protein phosphatase 1, regulatory (inhibitor) subunit 12A	M130/MBS/MYPT1	PPH18472C
Hs.677346	NM_002481	PPP1R12B	Protein phosphatase 1, regulatory (inhibitor) subunit 12B	MYPT2/PP1bp55	PPH15402B
Hs.435512	NM_000944	PPP3CA	Protein phosphatase 3, catalytic subunit, alpha isozyme	CALN/CALNA/CALNA1/CCN1/CNA1/PPP2B	PPH01496A
Hs.500067	NM_021132	PPP3CB	Protein phosphatase 3, catalytic subunit, beta isozyme	CALNA2/CALNB/CNA2/PP2Bbeta	PPH01495B
Hs.413812	NM_006908	RAC1	Ras-related C3 botulinum toxin substrate 1 (rho family, small GTP binding protein Rac1)	MIG5/Rac-1/TC-25/p21-Rac1	PPH00733F
Hs.505469	NM_013277	RACGAP1	Rac GTPase activating protein 1	CYK4/HsCYK-4/ID-GAP/MgcRacGAP	PPH02149A
Hs.263671	NM_002906	RDX	Radixin	DFNB24	PPH02808A
Hs.247077	NM_001664	RHOA	Ras homolog gene family, member A	ARH12/ARHA/RHO12/RHOH12	PPH00305G
Hs.306307	NM_005406	ROCK1	Rho-associated, coiled-coil containing protein kinase 1	P160ROCK/ROCK-I	PPH01966C
Hs.199763	NM_018984	SSH1	Slingshot homolog 1 (Drosophila)	SSH1L	PPH09650B
Hs.654754	NM_033389	SSH2	Slingshot homolog 2 (Drosophila)	SSH-2/SSH-2L	PPH08582B
Hs.209983	NM_005563	STMN1	Stathmin 1	C1orf215/LAP18/Lag/OP18/PP17/PP19/PR22/SMN	PPH14448B
Hs.517228	NM_003253	TIAM1	T-cell lymphoma invasion and metastasis 1	-	PPH05973F
Hs.515469	NM_003370	VASP	Vasodilator-stimulated phosphoprotein	-	PPH19132B
Hs.2157	NM_000377	WAS	Wiskott-Aldrich syndrome (eczema-thrombocytopenia)	IMD2/SCNX/THC/THC1/WASP/WASPA	PPH07123A
Hs.75850	NM_003931	WASF1	WAS protein family, member 1	SCAR1/WAVE/WAVE1	PPH09998A
Hs.143728	NM_003941	WASL	Wiskott-Aldrich syndrome-like	N-WASP/NWASP/WASPB	PPH13819A
Hs.520640	NM_001101	ACTB	Actin, beta	BRWS1/PS1TP5BP1	PPH00073G
Hs.534255	NM_004048	B2M	Beta-2-microglobulin	-	PPH01094E
Hs.592355	NM_002046	GAPDH	Glyceraldehyde-3-phosphate dehydrogenase	G3PD/GAPD/HEL-S-162eP	PPH00150F
Hs.412707	NM_000194	HPRT1	Hypoxanthine phosphoribosyltransferase 1	HGPRT/HPRT	PPH01018C
Hs.546285	NM_001002	RPLP0	Ribosomal protein, large, P0	L10E/LP0/P0/PRLP0/RPP0	PPH21138F
N/A	SA_00105	HGDC	Human Genomic DNA Contamination	HIGX1A	
N/A	SA_00104	RTC	Reverse Transcription Control	RTC	PPX63340A
N/A	SA_00104	RTC	Reverse Transcription Control	RTC	PPX63340A
N/A	SA_00104	RTC	Reverse Transcription Control	RTC	PPX63340A
N/A	SA_00103	PPC	Positive PCR Control	PPC	
N/A	SA_00103	PPC	Positive PCR Control	PPC	
N/A	SA_00103	PPC	Positive PCR Control	PPC	

B: HMN Autophagy Array
Catalog #: PAHS-084Z

Unigene	Refseq	Symbol	Description	Gname	RT2 Catalog
Hs.525622	NM_005163	AKT1	V-akt murine thymoma viral oncogene homolog 1	AKT/CWS6/PKB/PKB-ALPHA/PRKBA/RAC/RAC-ALPHA	PPH00088B
Hs.654644	NM_017749	AMBRA1	Autophagy/beclin-1 regulator 1	DCAF3/WDR94	PPH17960B
Hs.434980	NM_000484	APP	Amyloid beta (A4) precursor protein	AAA/ABETA/ABPP/AD1/APPI/CTFgamma/CVAP/PN-II/PN2	PPH05947A
Hs.713698	NM_031482	ATG10	ATG10 autophagy related 10 homolog (S. cerevisiae)	APG10/APG10L/pp12616	PPH19514G
Hs.264482	NM_004707	ATG12	ATG12 autophagy related 12 homolog (S. cerevisiae)	APG12/APG12L/FBR93/HAPG12	PPH15326A
Hs.529322	NM_017974	ATG16L1	ATG16 autophagy related 16-like 1 (S. cerevisiae)	APG16L/ATG16A/ATG16L/IBD10/WDR30	PPH19850A
Hs.653186	NM_033388	ATG16L2	ATG16 autophagy related 16-like 2 (S. cerevisiae)	ATG16B/WDR80	PPH08292A
Hs.477126	NM_022488	ATG3	ATG3 autophagy related 3 homolog (S. cerevisiae)	APG3/APG3-LIKE/APG3L/PC3-96	PPH08669A
Hs.8763	NM_052936	ATG4A	ATG4 autophagy related 4 homolog A (S. cerevisiae)	APG4A/AUTL2	PPH07642A
Hs.283610	NM_178326	ATG4B	ATG4 autophagy related 4 homolog B (S. cerevisiae)	APG4B/AUTL1	PPH15916A
Hs.7353	NM_178221	ATG4C	ATG4 autophagy related 4 homolog C (S. cerevisiae)	APG4-C/APG4C/AUTL1/AUTL3	PPH07503A
Hs.512799	NM_032885	ATG4D	ATG4 autophagy related 4 homolog D (S. cerevisiae)	APG4-D/APG4D/AUTL4	PPH23414A
Hs.486063	NM_004849	ATG5	ATG5 autophagy related 5 homolog (S. cerevisiae)	APG5/APG5-LIKE/APG5L/ASP/hAPG5	PPH07779A
Hs.740389	NM_006395	ATG7	ATG7 autophagy related 7 homolog (S. cerevisiae)	APG7-LIKE/APG7L/GSA7	PPH15687C
Hs.323363	NM_024085	ATG9A	ATG9 autophagy related 9 homolog A (S. cerevisiae)	APG9L1/MGD3208/mATG9	PPH16921A
Hs.707300	NM_173681	ATG9B	ATG9 autophagy related 9 homolog B (S. cerevisiae)	APG9L2/NOS3AS/SONE	PPH15659F
Hs.370254	NM_004322	BAD	BCL2-associated agonist of cell death	BBC2/BCL2L8	PPH00075C
Hs.485139	NM_001188	BAK1	BCL2-antagonist/killer 1	BAK/BAK-LIKE/BCL2L7/CDN1	PPH00077F
Hs.624291	NM_004324	BAX	BCL2-associated X protein	BCL2L4	PPH00078B
Hs.150749	NM_000633	BCL2	B-cell CLL/lymphoma 2	Bcl-2/PPP1R50	PPH00079B
Hs.516966	NM_138578	BCL2L1	BCL2-like 1	BCL-XL/S/BCL2L/BCLX/BCLXL/BCLXS/Bcl-X/PPP1R52/bcl-xL/bcl-xS	PPH00082C
Hs.716464	NM_003766	BECN1	Beclin 1, autophagy related	ATG6/VPS30/beclin1	PPH05670B
Hs.517145	NM_001196	BID	BH3 interacting domain death agonist	FP497	PPH00084C
Hs.144873	NM_004052	BNIP3	BCL2/adenovirus E1B 19kDa interacting protein 3	NIP3	PPH00301C
Hs.141125	NM_004346	CASP3	Caspase 3, apoptosis-related cysteine peptidase	CPP32/CPP32B/SCA-1	PPH00107C
Hs.599762	NM_001228	CASP8	Caspase 8, apoptosis-related cysteine peptidase	ALPS2B/CAP4/Casp-8/FLICE/MACH/MCH5	PPH00359F
Hs.238990	NM_004064	CDKN1B	Cyclin-dependent kinase inhibitor 1B (p27, Kip1)	CDKN4/KIP1/MEN1B/MEN4/P27KIP1	PPH00212C
Hs.512599	NM_000077	CDKN2A	Cyclin-dependent kinase inhibitor 2A (melanoma, p16, inhibits CDK4)	ARF/CDK4I/CDKN2/CMM2/INK4/INK4A/MLM/MTS-1/MTS1/P14/P14ARF/P16/P16-INK4A/P16INK4/P16INK4A/P19/P19ARF/TP16	PPH00207C
Hs.534667	NM_000086	CLN3	Ceroid-lipofuscinosis, neuronal 3	BTS/JNCL	PPH14111A
Hs.520898	NM_001908	CTSB	Cathepsin B	APPS/CPSB	PPH01053F
Hs.654447	NM_001909	CTSD	Cathepsin D	CLN10/CPSD/HEL-S-130P	PPH00112F
Hs.181301	NM_004079	CTSS	Cathepsin S	-	PPH06134A
Hs.593413	NM_003467	CXCR4	Chemokine (C-X-C motif) receptor 4	CD184/D2S201E/FB22/HM89/HSY3RR/LAP-3/LAP3/LCR1/LESTR/NPY3R/NPYR/NPYRL/NPYY3R/WHIM/WHIMS	PPH00621A
Hs.380277	NM_004938	DAPK1	Death-associated protein kinase 1	DAPK	PPH00273F
Hs.525634	NM_018370	DRAM1	DNA-damage regulated autophagy modulator 1	DRAM	PPH19768F
Hs.485606	NM_178454	DRAM2	DNA-damage regulated autophagy modulator 2	PRO180/TMEM77/WWFQ154	PPH14917A
Hs.591589	NM_004836	EIF2AK3	Eukaryotic translation initiation factor 2-alpha kinase 3	PEK/PERK/WRS	PPH10874A
Hs.433750	NM_182917	EIF4G1	Eukaryotic translation initiation factor 4 gamma, 1	EIF-4G1/EIF4F/EIF4G/EIF4GI/P220/PARK18	PPH20223A
Hs.744830	NM_000125	ESR1	Estrogen receptor 1	ER/ESR/ESRA/ESTRR/Era/NR3A1	PPH01001A
Hs.86131	NM_003824	FADD	Fas (TNFRSF6)-associated via death domain	GIG3/MORT1	PPH00367A
Hs.667309	NM_000043	FAS	Fas (TNF receptor superfamily, member 6)	ALPS1A/APO-1/APT1/CD95/FAS1/FASTM/TNFRSF6	PPH00141B
Hs.1437	NM_000152	GAA	Glucosidase, alpha; acid	LYAG	PPH07077F
Hs.647421	NM_007278	GABARAP	GABA(A) receptor-associated protein	ATG8A/GABARAP-a/MM46	PPH60029B
Hs.524250	NM_031412	GABARAPL1	GABA(A) receptor-associated protein like 1	APG8-LIKE/APG8L/ATG8/ATG8B/ATG8L/GEC1	PPH17234A
Hs.461379	NM_007285	GABARAPL2	GABA(A) receptor-associated protein-like 2	ATG8/ATG8C/GATE-16/GATE16/GEF-2/GEF2	PPH07417A
Hs.88556	NM_004964	HDAC1	Histone deacetylase 1	GON-10/HD1/RPD3/RPD3L1	PPH01735F
Hs.6764	NM_006044	HDAC6	Histone deacetylase 6	CPBHM/HD6/JM21/PPP1R90	PPH01475B
Hs.661056	NM_004712	HGS	Hepatocyte growth factor-regulated tyrosine kinase substrate	HRS	PPH19788A
Hs.525600	NM_001017963	HSP90AA1	Heat shock protein 90kDa alpha (cytosolic), class A member 1	EL52/HSP86/HSP89A/HSP90A/HSP90N/HSPC1/HSPCA/HSPCAL1/HSPCAL4/HSPN/Hsp89/Hsp90/LAP-2/LAP2	PPH63391B
Hs.180414	NM_006597	HSPA8	Heat shock 70kDa protein 8	HEL-33/HEL-S-72p/HSC54/HSC70/HSC71/HSP71/HSP73/HSPA10/LAP-1/LAP1/NIP71	PPH01211B
Hs.518450	NM_002111	HTT	Huntingtin	HD/IT15	PPH05750F
Hs.856	NM_000619	IFNG	Interferon, gamma	IFG/IFI	PPH00380C
Hs.160562	NM_000618	IGF1	Insulin-like growth factor 1 (somatomedin C)	IGF-I/IGFI/MGF	PPH00167C
Hs.700350	NM_000207	INS	Insulin	IDDM/IDDM1/IDDM2/ILPR/IRDN/MODY10	PPH00561C
Hs.519680	NM_001145805	IRGM	Immunity-related GTPase family, M	IFI1/IRGM1/LRG-47/LRG47	PPH58303E
Hs.494419	NM_005561	LAMP1	Lysosomal-associated membrane protein 1	CD107a/LAMPA/LGP120	PPH05794G
Hs.632273	NM_181509	MAP1LC3A	Microtubule-associated protein 1 light chain 3 alpha	ATG8E/LC3/LC3A/MAP1ALC3/MAP1BLC3	PPH19436A
Hs.356061	NM_022818	MAP1LC3B	Microtubule-associated protein 1 light chain 3 beta	ATG8F/LC3B/MAP1A/1BLC3/MAP1LC3B-a	PPH17765B
Hs.485233	NM_001315	MAPK14	Mitogen-activated protein kinase 14	CSBP/CSBP1/CSBP2/CSPB1/EXIP/Mxi2/PRKM14/PRKM15/RK/SAPK2A/p38/p38ALPHA	PPH00750B
Hs.138211	NM_002750	MAPK8	Mitogen-activated protein kinase 8	JNK/JNK-46/JNK1/JNK1A2/JNK21B1/2/PRKM8/SAPK1/SAPK1c	PPH00720B
Hs.338207	NM_004958	MTOR	Mechanistic target of rapamycin (serine/threonine kinase)	FRAP/FRAP1/FRAP2/RAFT1/RAPT1	PPH02311D
Hs.618430	NM_003998	NFKB1	Nuclear factor of kappa light polypeptide gene enhancer in B-cells 1	EBP-1/KBF1/NF-kB1/NF-kappa-B/NF-kappaB/NFKB-p105/NFKB-p50/NFkappaB/p105/p50	PPH00204F
Hs.464779	NM_000271	NPC1	Niemann-Pick disease, type C1	NPC	PPH06357A
Hs.656958	NM_002647	PIK3C3	Phosphoinositide-3-kinase, class 3	VPS34/Vps34/hVps34	PPH19820A
Hs.32942	NM_002649	PIK3CG	Phosphoinositide-3-kinase, catalytic, gamma polypeptide	PI3CG/PI3K/PI3Kgamma/PIK3/p110gamma/p120-PI3K	PPH02226A
Hs.149032	NM_014602	PIK3R4	Phosphoinositide-3-kinase, regulatory subunit 4	VPS15/p150	PPH16635A
Hs.43322	NM_006251	PRKAA1	Protein kinase, AMP-activated, alpha 1 catalytic subunit	AMPK/AMPKa1	PPH00043B
Hs.729457	NM_000314	PTEN	Phosphatase and tensin homolog	10q23del/BZS/CWS1/DEC/GLM2/MHAM/MMAC1/PTEN1/TEP1	PPH00327F
Hs.16258	NM_130781	RAB24	RAB24, member RAS oncogene family	-	PPH08089B
Hs.408528	NM_000321	RB1	Retinoblastoma 1	OSRC/PPP1R130/RB/p105-Rb/pRb/pp110	PPH00228F
Hs.422336	NM_005873	RGS19	Regulator of G-protein signaling 19	GAIP/RGSGAIP	PPH02525A
Hs.463642	NM_003161	RPS6KB1	Ribosomal protein S6 kinase, 70kDa, polypeptide 1	PS6K/S6K/S6K-beta-1/S6K1/STK14A/p70 S6KA/p70(S6K)-alpha/p70-S6K/p70-alpha	PPH00791F
Hs.21374	NM_000345	SNCA	Synuclein, alpha (non A4 component of amyloid precursor)	NACP/PARK1/PARK4/PD1	PPH05943G
Hs.587290	NM_003900	SQSTM1	Sequestosome 1	A170/FTDALS3/OSIL/PDB3/ZIP3/p60/p62/p62B	PPH02107A
Hs.645227	NM_000660	TGFB1	Transforming growth factor, beta 1	CED/DPD1/LAP/TGFB/TGFbeta	PPH00508A
Hs.517033	NM_004613	TGM2	Transglutaminase 2 (C polypeptide, protein-glutamine-gamma-glutamyltransferase)	G-ALPHA-h/GNAH/HEL-S-45/TG2/TGC	PPH23362A
Hs.99439	NM_153015	TMEM74	Transmembrane protein 74	NET36	PPH10786B
Hs.241570	NM_000594	TNF	Tumor necrosis factor	DIF/TNF-alpha/TNFA/TNFSF2	PPH00341F
Hs.478275	NM_003810	TNFSF10	Tumor necrosis factor (ligand) superfamily, member 10	APO2L/Apo-2L/CD253/TL2/TRAIL	PPH00242F
Hs.437460	NM_000546	TP53	Tumor protein p53	BCC7/LFS1/P53/TRP53	PPH00213F
Hs.47061	NM_003565	ULK1	Unc-51-like kinase 1 (C. elegans)	ATG1/ATG1A/UNC51/Unc51.1/hATG1	PPH09320A
Hs.168762	NM_014683	ULK2	Unc-51-like kinase 2 (C. elegans)	ATG1B/Unc51.2	PPH13378A
Hs.202470	NM_003369	UVRAG	UV radiation resistance associated gene	DHTX/VPS38/p63	PPH07910B
Hs.463964	NM_017983	WIPI1	WD repeat domain, phosphoinositide interacting 1	ATG18/ATG18A/WIPI49	PPH07685A
Hs.520640	NM_001101	ACTB	Actin, beta	BRWS1/PS1TP5BP1	PPH00073G
Hs.534255	NM_004048	B2M	Beta-2-microglobulin	-	PPH01094E
Hs.592355	NM_002046	GAPDH	Glyceraldehyde-3-phosphate dehydrogenase	G3PD/GAPD/HEL-S-162eP	PPH00150F
Hs.412707	NM_000194	HPRT1	Hypoxanthine phosphoribosyltransferase 1	HGPRT/HPRT	PPH01018C
Hs.546285	NM_001002	RPLP0	Ribosomal protein, large, P0	L10E/LP0/P0/PRLP0/RPP0	PPH21138F
N/A	SA_00105	HGDC	Human Genomic DNA Contamination	HIGX1A	
N/A	SA_00104	RTC	Reverse Transcription Control	RTC	PPX63340A
N/A	SA_00104	RTC	Reverse Transcription Control	RTC	PPX63340A
N/A	SA_00104	RTC	Reverse Transcription Control	RTC	PPX63340A
N/A	SA_00103	PPC	Positive PCR Control	PPC	
N/A	SA_00103	PPC	Positive PCR Control	PPC	
N/A	SA_00103	PPC	Positive PCR Control	PPC	

Supplemental Figure S3

A

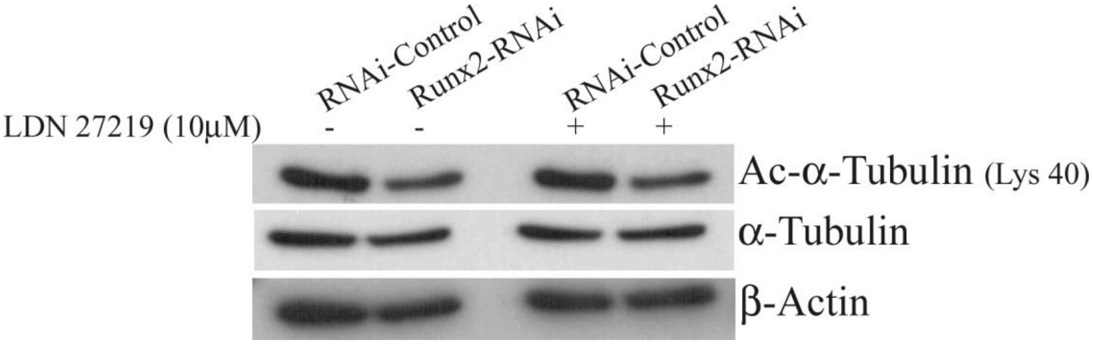

B

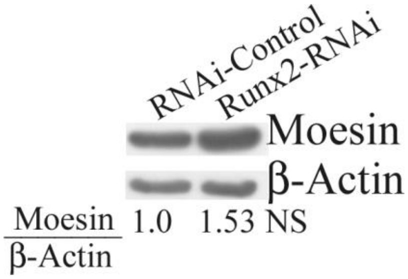

Supplemental Figure S4: Original Western Blots

Figure 3A

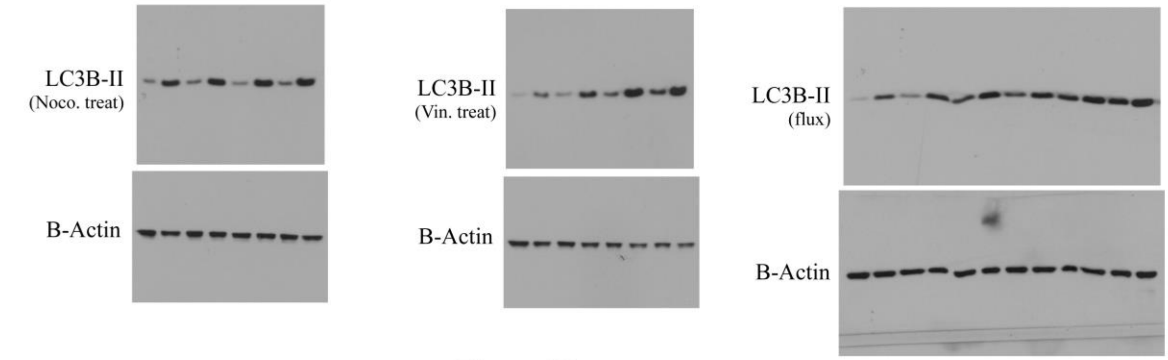

Figure 3B

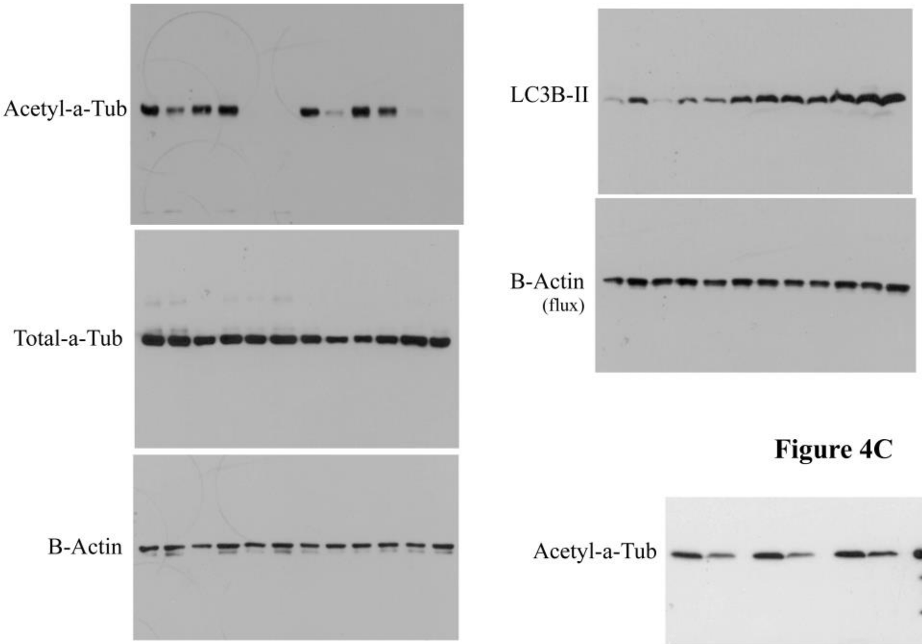

Figure 3C

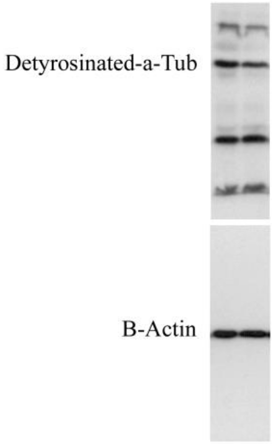

Figure 4B

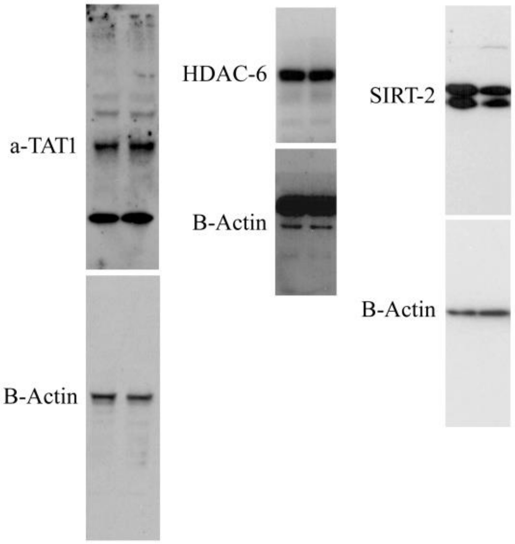

Figure 4C

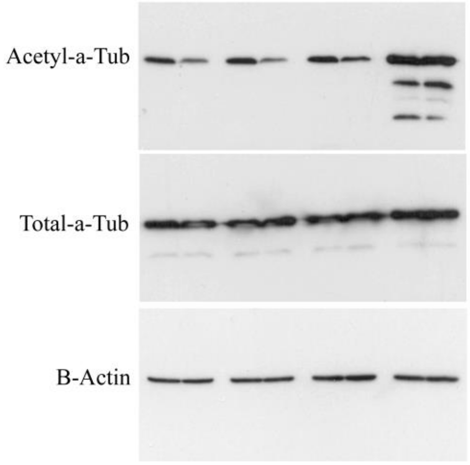

Figure 5C

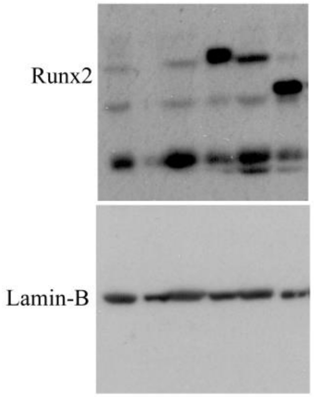

Figure 6A

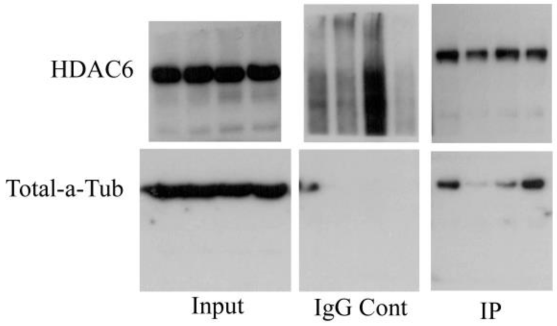

Figure 6B

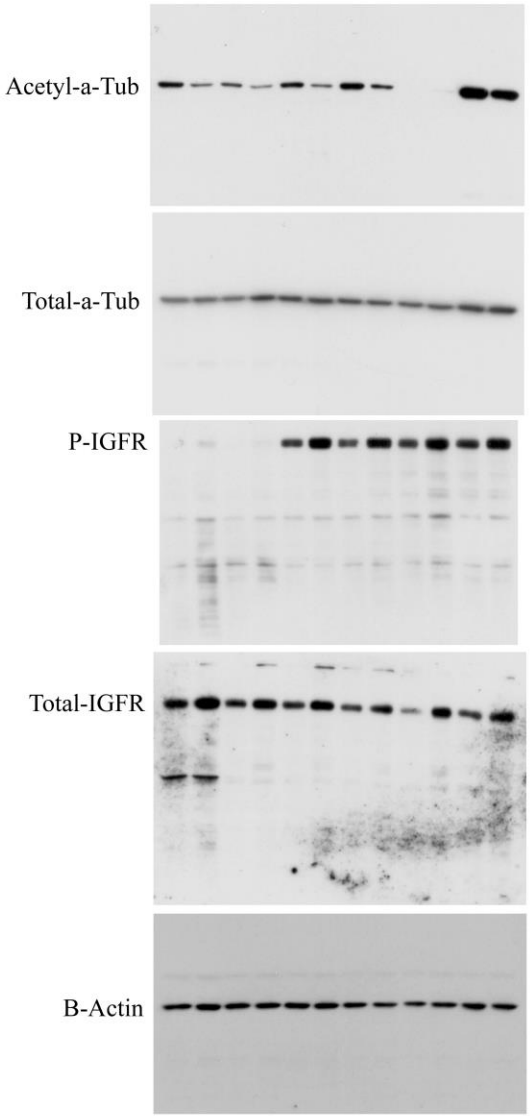

Figure 6C

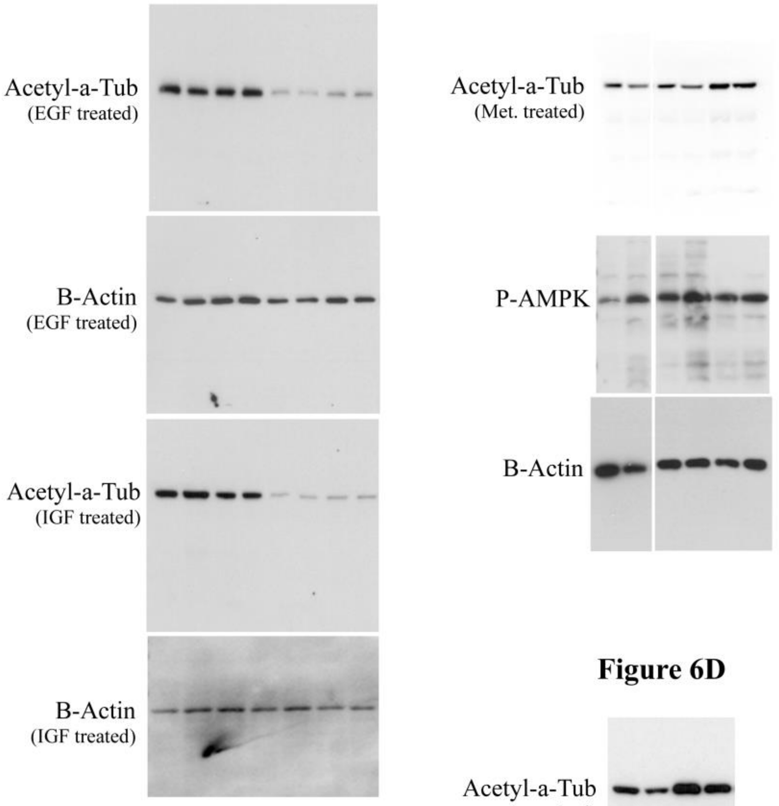

Figure 6D

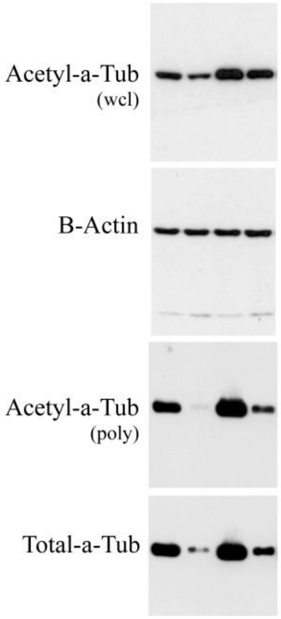

Supplement: Supplementary file 1 [file cancers-14-03436-s001.zip › cancers-1786519-supplementary.pdf]
